# Paradoxical Elevation of Parathyroid Hormone Levels following Preoperative Native Vitamin D Supplementation in a Patient with Graves’ Disease Undergoing Total Thyroidectomy: A Case Report

**DOI:** 10.70352/scrj.cr.25-0465

**Published:** 2025-10-30

**Authors:** Hiroyuki Yamashita, Hisakazu Shindo, Yusuke Mori, Daisuke Tatsushima, Takashi Fukuda, Seigo Tachibana, Hiroshi Takahashi, Yuji Nagayama, Shinya Sato

**Affiliations:** 1 Department of Surgery, Yamashita Thyroid Hospital, Fukuoka, Fukuoka, Japan; 2 Department of Endocrinology, Yamashita Thyroid Hospital, Fukuoka, Fukuoka, Japan

**Keywords:** vitamin D supplementation, secondary hyperparathyroidism, FGF23, total thyroidectomy, Graves’ disease

## Abstract

**INTRODUCTION:**

Hypocalcemia is a common complication after total thyroidectomy, particularly in patients with Graves’ disease and high bone turnover. Preoperative vitamin D deficiency is a known risk factor for postoperative hypocalcemia; however, the effects of vitamin D supplementation remain controversial. This is the first study to document a paradoxical increase in parathyroid hormone (PTH) levels after preoperative vitamin D supplementation in a patient undergoing total thyroidectomy.

**CASE PRESENTATION:**

We report the case of a 68-year-old woman with Graves’ disease and coexisting thyroid cancer who received native vitamin D (2000 IU/day) for 4 weeks before surgery. This unexpectedly resulted in a marked increase in PTH levels and bone formation markers, suggesting the worsening of secondary hyperparathyroidism. Interestingly, fibroblast growth factor 23 levels remained unchanged despite an increase in PTH and 1,25-dihydroxyvitamin D levels. Postoperatively, she developed transient hypoparathyroidism.

**CONCLUSIONS:**

Native vitamin D supplementation may, paradoxically, worsen secondary hyperparathyroidism in some patients, highlighting the need for careful preoperative metabolic assessment and individualized management strategies.

## Abbreviations

1,25(OH)_2_D
: 1,25-dihydroxyvitamin D
25(OH)D
: 25-hydroxyvitamin D
BAP
: bone-specific alkaline phosphatase
BMD
: bone mineral density
FGF23
: fibroblast growth factor 23
PTH
: parathyroid hormone
TRACP-5b
: tartrate-resistant acid phosphatase 5b

## INTRODUCTION

Hypoparathyroidism and hypocalcemia are well-documented complications of thyroidectomy. The risk of developing hypocalcemia post-thyroidectomy is influenced by various factors, including perioperative PTH levels, preoperative vitamin D deficiency, and potential parathyroid gland damage or removal during surgery.^1–3)^

Vitamin D is essential for calcium absorption and bone mineralization. Its deficiency impairs intestinal calcium absorption and leads to compensatory secondary hyperparathyroidism, which, in turn, increases bone turnover.^4)^ Therefore, vitamin D supplementation is widely recommended before thyroid surgery, particularly for patients at risk of developing hypocalcemia.^5,6)^ However, clinical studies have yielded inconsistent results.^7)^ These inconsistencies may be due to differences in the dosing regimens, baseline nutritional status, bone turnover activity, and timing relative to surgery.

Patients with Graves’ disease often exhibit accelerated bone metabolism owing to prolonged hyperthyroidism, resulting in reduced bone mineral density and increased calcium turnover.^8)^ This hypermetabolic bone state may alter the physiological responses to vitamin D repletion. Recently, attention has been directed toward the role of FGF23, a phosphaturic hormone secreted by osteocytes that regulates phosphate, calcium, and vitamin D homeostasis; it reduces PTH secretion and inhibits renal 1α-hydroxylase activity, thereby suppressing 1,25(OH)_2_D synthesis. Under normal conditions, increased PTH and 1,25(OH)_2_D levels stimulate FGF23 production via a feedback mechanism.^9)^ However, in the perioperative setting, particularly among patients with altered bone metabolism, the dynamics of FGF23 may be unpredictable and may affect calcium balance in ways that are not yet fully understood.

We previously reported that secondary hyperparathyroidism is a significant risk factor for developing postoperative hypocalcemia in patients undergoing total thyroidectomy for Graves’ disease.^2,10)^ Here, we present a case in which preoperative native vitamin D supplementation resulted in an unexpected increase in PTH levels, possibly due to an increased skeletal calcium demand, followed by transient hypocalcemia after surgery. This case contributes to our understanding of the variable effects of vitamin D supplementation and emphasizes the importance of individualized preoperative metabolic assessment.

## CASE PRESENTATION

A 68-year-old Japanese woman with a history of Graves’ disease was referred for surgical evaluation after being diagnosed with differentiated thyroid cancer. Despite ongoing antithyroid therapy (15 mg methimazole), the thyroid function was mildly elevated at the time of referral. She had no history of fractures or treatment for osteoporosis. Laboratory tests revealed a vitamin D deficiency (serum 25(OH)D: 10.9 ng/mL) and elevated bone turnover marker levels. Dual-energy X-ray absorptiometry revealed significantly reduced BMD, with a lumbar spine young value of 56%.

### Treatment and outcome

**Fig. 1:**
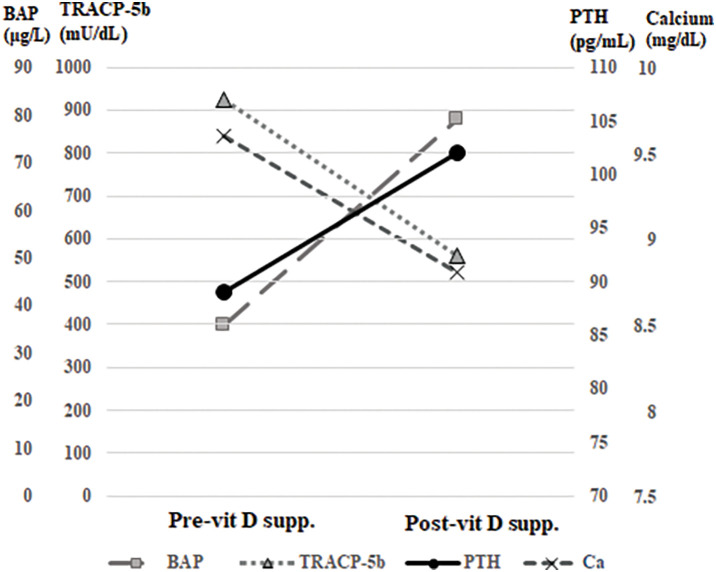
Changes in calcium, PTH, and bone markers during the clinical course. After vitamin D supplementation, secondary hyperparathyroidism worsened (serum calcium decreased and PTH increased), as did changes in bone metabolism (serum TRACP-5b decreased and BAP increased), indicating the predominance of bone formation. BAP, bone-specific alkaline phosphatase; Post-vit D supp., after vitamin D supplementation; Pre-vit D supp., before vitamin D supplementation; PTH, parathyroid hormone; TRACP-5b, tartrate-resistant acid phosphatase 5

To reduce the risk of developing postoperative hypocalcemia, the patient was administered native vitamin D (cholecalciferol) at a dose of 2000 IU per day for 4 weeks before surgery. Calcium was not supplemented during the study period. **Table 1** and **Fig. 1** show the results of calcium-related tests before and after vitamin D administration. Before supplementation, the patient's serum PTH level was 89 pg/mL, which increased to 102 pg/mL after 4 weeks. The levels of BAP, a marker of bone formation, increased from 33.8 to 62.8 μg/L. The levels of TRACP-5b, a marker of bone resorption, decreased from 923 to 562 mU/dL. This indicated a shift toward the dominance of bone formation (**Fig. 1**). There were no significant changes in FGF23 levels before or after vitamin D administration.

**Table 1:** Sequential changes in parameters after vitamin D supplementation and surgery

Parameter (reference range)	Pre-VitD	DOS am	POD 1	POD 3	POD 4w
25(OH)D (>30 ng/mL)	10.9	31.4			
PTH (10–15 pg/mL)	88.5	102	15.3		68.3
Calcium (8.8–10.1 mg/dL)	9.6	8.8	7.2	7.2	9.2
BAP (3.8–22.6 μg/L)	33.8	62.1			38.9
TRACP-5b (120–420 mU/dL)	923	629			661

25(OH)D, 25-hydroxyvitamin D; BAP, bone-specific alkaline phosphatase; DOS am, day of surgery morning; POD 1, postoperative day 1; POD 3, postoperative day 3; POD 4w, postoperative week 4; Pre-VitD, before Vitamin D supplementation; PTH, parathyroid hormone; TRACP-5b, tartrate-resistant acid phosphatase 5b

Total thyroidectomy with central neck lymph node dissection was performed; however, the tumor was adherent to the recurrent laryngeal nerve, resulting in the loss of the nerve integrity monitor response during the dissection procedure. Right recurrent laryngeal nerve paralysis was confirmed postoperatively by laryngeal fiber, but it healed after 3 months. On POD 1, the serum calcium level decreased from 8.8 mg/dL to 7.2 mg/dL, and the PTH level decreased from 102 pg/mL to 15.3 pg/mL, consistent with hypoparathyroidism. Although the patient showed no evident symptoms of tetany, treatment with 3 g of calcium lactate and 2 µg of alfacalcidol orally per day was initiated. Over the following 4 weeks, her serum calcium and PTH levels normalized, and serum BAP level decreased to pre-vitamin D administration level. However, her TRACP-5b level remained elevated (**Table 1**). Three weeks after surgery, the patient discontinued all supplements. At 9 months postoperatively, she remained well without recurrence and required no oral vitamin D or calcium supplementation.

## DISCUSSION

This case was part of a pilot study in which patients received vitamin D supplementation before surgery to prevent hypocalcemia after total thyroidectomy. Although high-dose vitamin D loading is commonly practiced in some countries,^5,6)^ it is not routinely recommended in Japan. Therefore, we investigated whether daily supplementation with 2000 IU of native vitamin D for at least 3 weeks would correct vitamin D deficiency. Calcium supplementation was intentionally excluded from the protocol due to concerns regarding patient adherence.

To the best of our knowledge, no previous report has documented a paradoxical increase in PTH levels after preoperative vitamin D supplementation in patients undergoing total thyroidectomy. Although vitamin D repletion is typically linked to PTH suppression, as supported by previous studies including the recent systematic review and meta-analysis by Khodadadiyan et al.,^11)^ this case shows that, under certain physiological conditions such as pre-existing low bone mineral density, active bone turnover, and insufficient calcium reserves, vitamin D supplementation can instead enhance skeletal calcium uptake and stimulate an increase in PTH levels, leading to secondary hyperparathyroidism.

In this case, 4 weeks after natural vitamin D supplementation, the 25(OH)D levels increased to levels within the normal range. It appeared to promote bone formation by increasing calcium utilization by osteoblasts, which subsequently increased PTH levels. This mechanism is supported by the observed shift in bone turnover marker levels. The BAP levels increased significantly, whereas the TRACP-5b levels decreased. This suggests that vitamin D supplementation activated bone formation. Owing to the patient's low bone mass and pre-existing high bone turnover rate caused by Graves’ disease, the skeletal system likely acted as a calcium reservoir during vitamin D-induced osteoblast activation. This calcium sequestration likely leads to a subclinical decrease in serum calcium levels, resulting in a reactive increase in PTH levels. Despite PTH elevation, the FGF23 levels remained unchanged. Under normal conditions, both PTH and 1,25(OH)_2_D stimulate FGF23 production. One possible explanation for the lack of an FGF23 response is that the suppression of FGF23 may function as a compensatory mechanism to prevent further reductions in serum calcium levels in the setting of secondary hyperparathyroidism and increased skeletal demand in Graves’ disease. Considering that FGF23 inhibits 1,25(OH)_2_D synthesis, its overexpression can exacerbate hypocalcemia. Thus, the reduced FGF23 response observed in this study may be an adaptive physiological response that preserves calcium homeostasis in a metabolically vulnerable state.

Additionally, excess thyroid hormones alter calcium metabolism and enhance bone turnover, which may further modulate the response to vitamin D supplementation.^8)^ This case suggests that even modest vitamin D supplementation may influence perioperative calcium metabolism, although the responses may vary depending on factors such as bone turnover status and hormonal feedback mechanisms.

Notably, calcium was not administered along with vitamin D before surgery for the aforementioned reasons. Calcium supplementation is essential to buffer increased skeletal demand during vitamin D-driven bone remodeling in patients with low BMD or high bone turnover. Our previous study highlighted the role of secondary hyperparathyroidism as a predictor of postoperative hypocalcemia,^2,10)^ and this case illustrates how vitamin D supplementation without calcium support can increase the risk of hypocalcemia.

## CONCLUSIONS

This case underscores the importance of a personalized approach for the preoperative management of patients undergoing thyroidectomy, particularly those with Graves’ disease and a low BMD. Although vitamin D supplementation is generally beneficial, it may, in some cases, promote bone formation to the point of increasing skeletal calcium requirements and temporarily worsen calcium balance. Combined calcium and vitamin D supplementation, as well as preoperative assessment of bone turnover and parathyroid reserve, may mitigate this risk. Further research is needed to clarify the optimal preoperative strategies as well as the roles of FGF23, bone markers, and thyroid status in calcium homeostasis.

## DECLARATIONS

### Availability of data and materials

Data sharing is not applicable to this article because no datasets were generated or analyzed in the current study.

## References

[1] Mihai R, Thakker RV. Management of endocrine disease: postsurgical hypoparathyroidism: current treatments and future prospects for parathyroid allotransplantation. Eur J Endocrinol 2021; 184: R165–75. 33599211 10.1530/EJE-20-1367 PMC8052514

[2] Yamashita H, Sato S, Shindo H, et al. A prospective cross-sectional study on hypocalcemia after total thyroidectomy in patients with Graves’ disease: insights on secondary hyperparathyroidism. Surg Today 2024; 54: 1058–66. 38635056 10.1007/s00595-024-02848-4

[3] Maheshwari M, Khan IA. Risk factors for transient and permanent hypoparathyroidism following thyroidectomy: a comprehensive review. Cureus 2024; 16: e66551. 39258042 10.7759/cureus.66551 PMC11383864

[4] Voulgaridou G, Papadopoulou SK, Detopoulou P, et al. Vitamin D and calcium in osteoporosis, and the role of bone turnover markers: a narrative review of recent data from RCTs. Diseases 2023; 11: 29. 36810543 10.3390/diseases11010029 PMC9944083

[5] Kannan T, Foster Y, Ho DJ, et al. Post-operative permanent hypoparathyroidism and preoperative vitamin D prophylaxis. J Clin Med 2021; 10: 442. 33498810 10.3390/jcm10030442 PMC7865725

[6] Sessa L, De Crea C, Zotta F, et al. Post-thyroidectomy hypocalcemia: is a routine preferable over a selective supplementation? Am J Surg 2022; 223: 1126–31. 34711410 10.1016/j.amjsurg.2021.10.015

[7] Casey C, Hopkins D. The role of preoperative vitamin D and calcium in preventing post-thyroidectomy hypocalcaemia: a systematic review. Eur Arch Otorhinolaryngol 2023; 280: 1555–63. 36542113 10.1007/s00405-022-07791-z

[8] Khamisi S, Lundqvist M, Rasmusson AJ, et al. Vitamin D and bone metabolism in Graves’ disease: a prospective study. J Endocrinol Invest 2023; 46: 425–33. 36166168 10.1007/s40618-022-01927-y PMC9859854

[9] Latic N, Erben RG. FGF23 and vitamin D metabolism. JBMR Plus 2021; 5: e10558. 34950827 10.1002/jbm4.10558 PMC8674776

[10] Yamashita H, Mori Y, Sato S, et al. Significant role of 1,25-dihydroxyvitamin D on serum calcium levels after total thyroidectomy: a prospective cohort study. Front Endocrinol (Lausanne) 2024; 15: 1360464. 38803480 10.3389/fendo.2024.1360464 PMC11128608

[11] Khodadadiyan A, Rahmanian M, Shekouh D, et al. Evaluating the effect of vitamin D supplementation on serum levels of 25-hydroxy vitamin D, 1,25-dihydroxy vitamin D, parathyroid hormone and renin-angiotensin–aldosterone system: a systematic review and meta-analysis of clinical trials. BMC Nutr 2023; 15; 9: 132. 10.1186/s40795-023-00786-x PMC10652523 37968749

